# About an observation of coccydynia treated by surgery

**DOI:** 10.11604/pamj.2020.36.135.19904

**Published:** 2020-06-26

**Authors:** Noukhoum Koné

**Affiliations:** 1 Service de Neurochirurgie, Centre Hospitalier de Kiffa, Kiffa, Mauritanie

**Keywords:** Coccydynia, coccygiectomy, coccyx

## Image in medicine

Coccygodynia is pain located in coccyx. Factors that increase are stay position and stand up. Trauma seems to be his first cause, it can be falled in a sitting position, post-delivery or repeated micro-trauma. Therapeutic management depends on the mechanism involved and relies on manual treatments, infiltrations, even surgery, with good results and a success rate of about 90%. We report the case of a 23-year-old patient with disabling coccygeal pain, progressively worsening, following a fall of a floor 4 meters high, the patient sitting with reception on the buttocks. The pains have a repercussion on the sphincter function with the installation of a rather severe constipation. The examination finds an exquisite pain with regard to the sacrum on palpation without any sign of disco-radicular conflict and the skin facing is normal. The patient has tried everything in terms of conservative treatment including manipulations. The sacro-coccygeal CT shows in sagittal section (A) a dislocation of the entire coccyx compared to the 5^th^ sacred piece. The incision arcuate (B) was preferred over the median vertical incision in the intergluteal fold closer to the anus with a non-nefiable infectious risk, ranging from 6.15 to 16.6% depending on the data from the terature. Excision of the coccyx (C) was performed under general anesthesia, the patient installed in ventral decubitus. The clinical evolution was significantly favorable.

**Figure 1:**
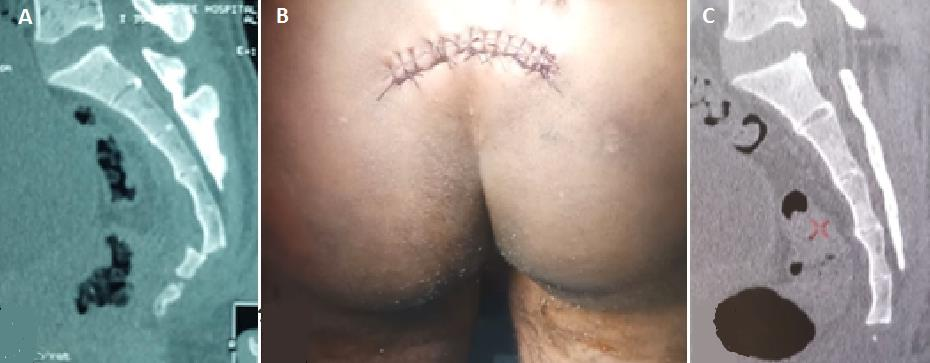
(A) sacro-coccygeal CT scan showing in sagittal section, a dislocation of the entire coccyx compared to the 5^th^ sacred piece; (B) surgical approach; (C) an excision of the coccyx

